# Ethyl 6-(4-cyclo­propyl-1*H*-1,2,3-triazol-1-yl)pyridine-3-carboxyl­ate

**DOI:** 10.1107/S1600536813034673

**Published:** 2014-01-15

**Authors:** Muhammad Naeem Ahmed, Khawaja Ansar Yasin, M. Nawaz Tahir, Asma Bibi, Hina Andleeb

**Affiliations:** a Department of Chemistry, The University of Azad Jammu and Kashmir, Muzaffarabad 13100, Pakistan; b University of Sargodha, Department of Physics, Sargodha, Pakistan; c Department of Chemistry, Quaid-e-Azam University, Islamabad 45320, Pakistan

## Abstract

In the title compound, C_13_H_14_N_4_O_2_, which has approximate mirror symmetry, the dihedral angles between the triazole ring and the cyclo­propane and pyridine rings are 87.1 (2) and 7.60 (9)°, respectively. In the crystal, inversion dimers linked by pairs of both C—H⋯N and C—H⋯O inter­actions generate *R*_2_^2^(6) and *R*_2_^2^(18) loops, respectively. Further C—H⋯N inter­actions form *R*_2_^2^(10) loops and link the dimers into [110] chains.

## Related literature   

For background to triazoles, see: Kiselyova *et al.* (2009 ▶). For the synthesis, see: Zhang *et al.* (2012 ▶).
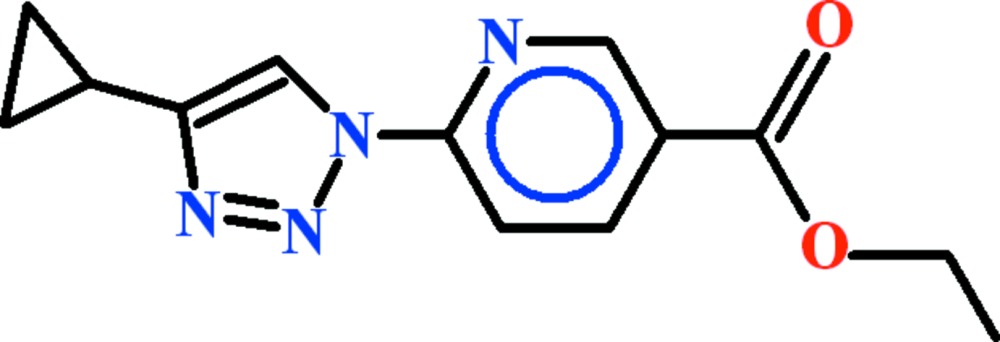


## Experimental   

### 

#### Crystal data  

C_13_H_14_N_4_O_2_
*M* *_r_* = 258.28
Triclinic, 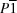

*a* = 7.1565 (6) Å
*b* = 9.3521 (8) Å
*c* = 9.8747 (9) Å
α = 85.722 (4)°
β = 81.422 (4)°
γ = 86.941 (4)°
*V* = 651.09 (10) Å^3^
*Z* = 2
Mo *K*α radiation
μ = 0.09 mm^−1^
*T* = 296 K
0.42 × 0.30 × 0.28 mm

#### Data collection  

Bruker Kappa APEXII CCD diffractometer
Absorption correction: multi-scan (*SADABS*; Bruker, 2005 ▶) *T*_min_ = 0.964, *T*_max_ = 0.975
8987 measured reflections
2484 independent reflections
1756 reflections with *I* > 2σ(*I*)
*R*_int_ = 0.029

#### Refinement  

*R*[*F* ^2^ > 2σ(*F* ^2^)] = 0.046
*wR*(*F* ^2^) = 0.137
*S* = 1.04
2484 reflections
173 parameters
H-atom parameters constrained
Δρ_max_ = 0.12 e Å^−3^
Δρ_min_ = −0.26 e Å^−3^

### 

Data collection: *APEX2* (Bruker, 2007 ▶); cell refinement: *SAINT* (Bruker, 2007 ▶); data reduction: *SAINT*; program(s) used to solve structure: *SHELXS97* (Sheldrick, 2008 ▶); program(s) used to refine structure: *SHELXL97* (Sheldrick, 2008 ▶); molecular graphics: *ORTEP-3 for Windows* (Farrugia, 2012 ▶) and *PLATON* (Spek, 2009 ▶); software used to prepare material for publication: *WinGX* (Farrugia, 2012 ▶) and *PLATON*.

## Supplementary Material

Crystal structure: contains datablock(s) global, I. DOI: 10.1107/S1600536813034673/hb7179sup1.cif

Structure factors: contains datablock(s) I. DOI: 10.1107/S1600536813034673/hb7179Isup2.hkl

Supporting information file. DOI: 10.1107/S1600536813034673/hb7179Isup3.cml

CCDC reference:

Additional supporting information:  crystallographic information; 3D view; checkCIF report

## Figures and Tables

**Table 1:** Hydrogen-bond geometry (Å, °)

*D*—H⋯*A*	*D*—H	H⋯*A*	*D*⋯*A*	*D*—H⋯*A*
C5—H5⋯N1^i^	0.93	2.60	3.375 (2)	141
C7—H7⋯N3^ii^	0.93	2.42	3.245 (2)	147
C9—H9⋯O2^i^	0.93	2.54	3.422 (2)	159

Symmetry codes: (i) 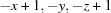; (ii) 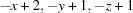.

**Fig. 1.**
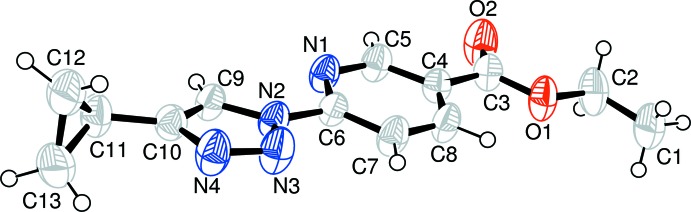
View of the title compound with displacement ellipsoids drawn at the 50% probability level.

**Fig. 2.**
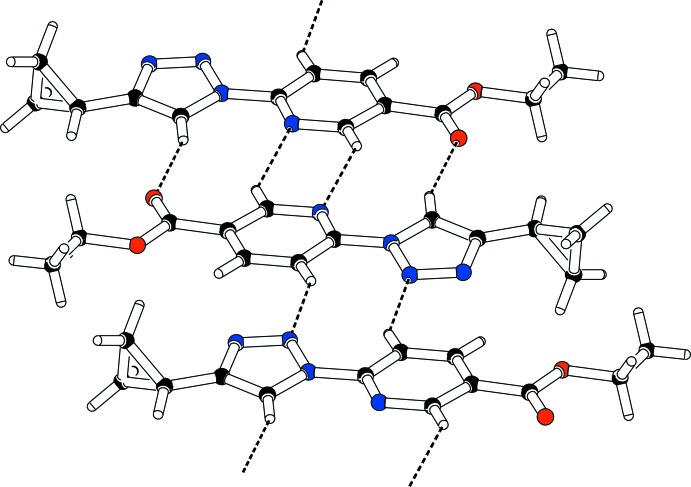
Partial packing diagram of the title compound showing that molecules form dimers and various ring motifs as parts of chains.

## supplementary crystallographic information

### 1. Comment

There are many derivatives of 1-(pyridine-2yl)-1,2,3-triazole that have been used as an intermediates in organic synthesis, or as ligands in coordination chemistry which have shown biologically important properties (Kiselyova *et al.*, 2009; Zhang *et al.*, 2012). As part of our studies in this area, we report here the structure of the title compound (I), (Fig. 1).

In (I) the pyridine ring A (C4–C8/N1) and 1, 2,3-triazol ring B (C9/C10/N2/N3/N4) are almost planar with r.m.s. deviations of 0.0004 Å and 0.0014 Å, respectively. The dihedral angle between A/B is 7.61 (12)°. The ethyl acetate moiety attached with pyridine ring is also close to planar with r.m.s. deviation of 0.0088 Å and is oriented at a dihedral angle of 4.21 (15)° with ring A. The cyclopropyl moiety is of course planar and makes adihedral angle of 87.12 (12)° with the triazole ring. The molecules form dimers due to inversion and *R*_2_^2^(6) an *R*_2_^2^(10) loops are formed due to H-bondings of C—H····N and C—H····O types (Table 1, Fig. 2). The dimers are linked into [110] chains forming *R*_2_^2^(10) ring motif due to C—H····N type of H-bonding.

### 2. Experimental

To a suspension of ethyl tetrazolo [1,5-a]pyridine-6-carboxylate (0.25 g, 1 mmol), copper acetate (0.018 g, 0.1 mmol) in THF (2 ml) was stirred for 10 min, to give a deep red color suspension. Ethynylcyclopropane (0.072 g, 1.1 mmol) was added and the reaction mixture was stirred at room temperature for 40 min. The residue was purified by column chromatography to give 84% of product as white solid. Colourless prisms of (I) were grown by slow evaporation of an ethanol: ethyl acetate solution at room temperature (m.p. 394 K).

### 3. Refinement

The H-atoms were positioned geometrically (C–H = 0.93–0.97 Å) and refined as riding with *U*_iso_(H) = *xU*_eq_(C), where *x* = 1.5 for methyl and *x* = 1.2 for other H-atoms.

### Crystal data

**Table:**

C_13_H_14_N_4_O_2_	*Z* = 2
*M**_r_* = 258.28	*F*(000) = 272
Triclinic, *P*1	*D*_x_ = 1.317 Mg m^−^^3^
*a* = 7.1565 (6) Å	Mo *K*α radiation, λ = 0.71073 Å
*b* = 9.3521 (8) Å	Cell parameters from 1756 reflections
*c* = 9.8747 (9) Å	θ = 2.1–26.0°
α = 85.722 (4)°	µ = 0.09 mm^−^^1^
β = 81.422 (4)°	*T* = 296 K
γ = 86.941 (4)°	Prism, colourless
*V* = 651.09 (10) Å^3^	0.42 × 0.30 × 0.28 mm

### Data collection

**Table:**

Bruker Kappa APEXII CCD diffractometer	2484 independent reflections
Radiation source: fine-focus sealed tube	1756 reflections with *I* > 2σ(*I*)
Graphite monochromator	*R*_int_ = 0.029
Detector resolution: 8.00 pixels mm^-1^	θ_max_ = 26.0°, θ_min_ = 2.1°
ω scans	*h* = −7→9
Absorption correction: multi-scan (*SADABS*; Bruker, 2005)	*k* = −11→11
*T*_min_ = 0.964, *T*_max_ = 0.975	*l* = −12→12
8987 measured reflections	

### Refinement

**Table:**

Refinement on *F*^2^	Primary atom site location: structure-invariant direct methods
Least-squares matrix: full	Secondary atom site location: difference Fourier map
*R*[*F*^2^ > 2σ(*F*^2^)] = 0.046	Hydrogen site location: inferred from neighbouring sites
*wR*(*F*^2^) = 0.137	H-atom parameters constrained
*S* = 1.04	*w* = 1/[σ^2^(*F*_o_^2^) + (0.0667*P*)^2^ + 0.1169*P*] where *P* = (*F*_o_^2^ + 2*F*_c_^2^)/3
2484 reflections	(Δ/σ)_max_ < 0.001
173 parameters	Δρ_max_ = 0.12 e Å^−^^3^
0 restraints	Δρ_min_ = −0.26 e Å^−^^3^

### Special details

**Table:**

Geometry. All e.s.d.'s (except the e.s.d. in the dihedral angle between two l.s. planes) are estimated using the full covariance matrix. The cell e.s.d.'s are taken into account individually in the estimation of e.s.d.'s in distances, angles and torsion angles; correlations between e.s.d.'s in cell parameters are only used when they are defined by crystal symmetry. An approximate (isotropic) treatment of cell e.s.d.'s is used for estimating e.s.d.'s involving l.s. planes.
Refinement. Refinement of *F*^2^ against ALL reflections. The weighted *R*-factor *wR* and goodness of fit *S* are based on *F*^2^, conventional *R*-factors *R* are based on *F*, with *F* set to zero for negative *F*^2^. The threshold expression of *F*^2^ > σ(*F*^2^) is used only for calculating *R*-factors(gt) *etc*. and is not relevant to the choice of reflections for refinement. *R*-factors based on *F*^2^ are statistically about twice as large as those based on *F*, and *R*-factors based on ALL data will be even larger.

### Fractional atomic coordinates and isotropic or equivalent isotropic displacement parameters (Å^2^)

**Table:**

	*x*	*y*	*z*	*U*_iso_*/*U*_eq_	
C1	0.2414 (3)	0.6459 (2)	0.0441 (2)	0.0859 (7)	
H1A	0.2315	0.7147	0.1125	0.129*	
H1B	0.1520	0.6722	−0.0179	0.129*	
H1C	0.3671	0.6441	−0.0061	0.129*	
C2	0.2005 (3)	0.5009 (2)	0.1119 (2)	0.0751 (6)	
H2A	0.0739	0.5012	0.1633	0.090*	
H2B	0.2098	0.4302	0.0439	0.090*	
C3	0.3330 (3)	0.33974 (18)	0.27255 (18)	0.0522 (5)	
C4	0.4902 (2)	0.31531 (17)	0.35605 (16)	0.0458 (4)	
C5	0.5060 (3)	0.18591 (18)	0.43204 (18)	0.0521 (5)	
H5	0.4154	0.1186	0.4303	0.062*	
C6	0.7698 (2)	0.25016 (17)	0.50747 (16)	0.0434 (4)	
C7	0.7690 (3)	0.38274 (18)	0.43583 (19)	0.0556 (5)	
H7	0.8617	0.4477	0.4394	0.067*	
C8	0.6251 (3)	0.41465 (18)	0.35887 (19)	0.0545 (5)	
H8	0.6188	0.5027	0.3090	0.065*	
C9	0.9478 (2)	0.08458 (17)	0.66058 (16)	0.0470 (4)	
H9	0.8839	0.0002	0.6628	0.056*	
C10	1.0960 (2)	0.10746 (18)	0.72627 (17)	0.0480 (4)	
C11	1.1936 (3)	0.0111 (2)	0.82036 (18)	0.0589 (5)	
H11	1.1577	−0.0892	0.8280	0.071*	
C12	1.2412 (3)	0.0654 (3)	0.9484 (2)	0.0734 (6)	
H12A	1.2100	0.1655	0.9646	0.088*	
H12B	1.2292	0.0006	1.0304	0.088*	
C13	1.3932 (3)	0.0332 (3)	0.8366 (2)	0.0763 (6)	
H13A	1.4747	−0.0514	0.8498	0.092*	
H13B	1.4554	0.1135	0.7840	0.092*	
N3	1.0348 (2)	0.30912 (16)	0.61267 (17)	0.0650 (5)	
N4	1.1459 (2)	0.24626 (17)	0.69538 (17)	0.0640 (5)	
N1	0.6440 (2)	0.15211 (14)	0.50782 (15)	0.0502 (4)	
N2	0.9131 (2)	0.21107 (14)	0.59114 (13)	0.0466 (4)	
O1	0.34012 (19)	0.46736 (13)	0.20353 (14)	0.0657 (4)	
O2	0.2151 (2)	0.25401 (15)	0.26590 (15)	0.0774 (5)	

### Atomic displacement parameters (Å^2^)

**Table:**

	*U*^11^	*U*^22^	*U*^33^	*U*^12^	*U*^13^	*U*^23^
C1	0.0925 (17)	0.0819 (16)	0.0814 (16)	0.0190 (13)	−0.0276 (13)	0.0204 (12)
C2	0.0823 (15)	0.0681 (13)	0.0825 (15)	0.0107 (11)	−0.0465 (12)	0.0050 (11)
C3	0.0573 (11)	0.0432 (9)	0.0584 (11)	−0.0001 (8)	−0.0188 (9)	0.0008 (8)
C4	0.0487 (10)	0.0421 (9)	0.0483 (10)	−0.0026 (7)	−0.0144 (8)	0.0011 (7)
C5	0.0559 (11)	0.0437 (9)	0.0606 (11)	−0.0134 (8)	−0.0224 (9)	0.0066 (8)
C6	0.0462 (9)	0.0407 (9)	0.0449 (9)	−0.0068 (7)	−0.0137 (7)	0.0048 (7)
C7	0.0581 (11)	0.0443 (9)	0.0680 (12)	−0.0169 (8)	−0.0240 (9)	0.0123 (8)
C8	0.0631 (11)	0.0400 (9)	0.0621 (11)	−0.0085 (8)	−0.0213 (9)	0.0140 (8)
C9	0.0543 (10)	0.0389 (9)	0.0490 (10)	−0.0059 (7)	−0.0149 (8)	0.0059 (7)
C10	0.0457 (10)	0.0507 (10)	0.0493 (10)	−0.0040 (8)	−0.0143 (8)	0.0019 (7)
C11	0.0604 (12)	0.0581 (11)	0.0621 (12)	−0.0009 (9)	−0.0283 (9)	0.0072 (9)
C12	0.0744 (14)	0.0934 (16)	0.0558 (12)	0.0060 (12)	−0.0259 (11)	−0.0001 (11)
C13	0.0583 (13)	0.1031 (17)	0.0695 (14)	0.0069 (11)	−0.0248 (11)	0.0038 (12)
N3	0.0659 (10)	0.0523 (9)	0.0836 (11)	−0.0223 (8)	−0.0369 (9)	0.0176 (8)
N4	0.0615 (10)	0.0589 (10)	0.0776 (11)	−0.0153 (8)	−0.0344 (8)	0.0137 (8)
N1	0.0543 (9)	0.0429 (8)	0.0570 (9)	−0.0127 (6)	−0.0219 (7)	0.0092 (6)
N2	0.0496 (8)	0.0417 (8)	0.0513 (8)	−0.0122 (6)	−0.0183 (6)	0.0073 (6)
O1	0.0711 (9)	0.0536 (8)	0.0781 (9)	−0.0014 (6)	−0.0386 (7)	0.0135 (6)
O2	0.0769 (10)	0.0619 (9)	0.1036 (11)	−0.0156 (8)	−0.0500 (9)	0.0114 (8)

### Geometric parameters (Å, º)

**Table:**

C1—C2	1.490 (3)	C7—H7	0.9300
C1—H1A	0.9600	C8—H8	0.9300
C1—H1B	0.9600	C9—N2	1.353 (2)
C1—H1C	0.9600	C9—C10	1.357 (2)
C2—O1	1.453 (2)	C9—H9	0.9300
C2—H2A	0.9700	C10—N4	1.365 (2)
C2—H2B	0.9700	C10—C11	1.471 (2)
C3—O2	1.206 (2)	C11—C13	1.488 (3)
C3—O1	1.329 (2)	C11—C12	1.489 (3)
C3—C4	1.490 (2)	C11—H11	0.9800
C4—C8	1.380 (2)	C12—C13	1.466 (3)
C4—C5	1.384 (2)	C12—H12A	0.9700
C5—N1	1.337 (2)	C12—H12B	0.9700
C5—H5	0.9300	C13—H13A	0.9700
C6—N1	1.318 (2)	C13—H13B	0.9700
C6—C7	1.381 (2)	N3—N4	1.310 (2)
C6—N2	1.427 (2)	N3—N2	1.347 (2)
C7—C8	1.376 (2)		
			
C2—C1—H1A	109.5	N2—C9—H9	127.5
C2—C1—H1B	109.5	C10—C9—H9	127.5
H1A—C1—H1B	109.5	C9—C10—N4	108.25 (15)
C2—C1—H1C	109.5	C9—C10—C11	130.37 (16)
H1A—C1—H1C	109.5	N4—C10—C11	121.34 (16)
H1B—C1—H1C	109.5	C10—C11—C13	120.71 (18)
O1—C2—C1	106.77 (18)	C10—C11—C12	119.78 (17)
O1—C2—H2A	110.4	C13—C11—C12	58.99 (13)
C1—C2—H2A	110.4	C10—C11—H11	115.3
O1—C2—H2B	110.4	C13—C11—H11	115.3
C1—C2—H2B	110.4	C12—C11—H11	115.3
H2A—C2—H2B	108.6	C13—C12—C11	60.47 (14)
O2—C3—O1	123.76 (17)	C13—C12—H12A	117.7
O2—C3—C4	124.61 (16)	C11—C12—H12A	117.7
O1—C3—C4	111.62 (15)	C13—C12—H12B	117.7
C8—C4—C5	117.89 (16)	C11—C12—H12B	117.7
C8—C4—C3	123.04 (15)	H12A—C12—H12B	114.8
C5—C4—C3	119.04 (15)	C12—C13—C11	60.54 (14)
N1—C5—C4	123.74 (16)	C12—C13—H13A	117.7
N1—C5—H5	118.1	C11—C13—H13A	117.7
C4—C5—H5	118.1	C12—C13—H13B	117.7
N1—C6—C7	125.18 (15)	C11—C13—H13B	117.7
N1—C6—N2	114.66 (14)	H13A—C13—H13B	114.8
C7—C6—N2	120.14 (15)	N4—N3—N2	107.00 (14)
C8—C7—C6	117.21 (16)	N3—N4—C10	109.09 (14)
C8—C7—H7	121.4	C6—N1—C5	116.32 (14)
C6—C7—H7	121.4	N3—N2—C9	110.61 (14)
C7—C8—C4	119.65 (15)	N3—N2—C6	119.87 (13)
C7—C8—H8	120.2	C9—N2—C6	129.50 (14)
C4—C8—H8	120.2	C3—O1—C2	116.97 (15)
N2—C9—C10	105.05 (14)		
			
O2—C3—C4—C8	178.53 (18)	C10—C11—C13—C12	108.5 (2)
O1—C3—C4—C8	−0.3 (2)	N2—N3—N4—C10	0.3 (2)
O2—C3—C4—C5	0.2 (3)	C9—C10—N4—N3	−0.4 (2)
O1—C3—C4—C5	−178.58 (15)	C11—C10—N4—N3	−178.48 (16)
C8—C4—C5—N1	0.1 (3)	C7—C6—N1—C5	0.1 (3)
C3—C4—C5—N1	178.47 (16)	N2—C6—N1—C5	179.08 (14)
N1—C6—C7—C8	0.0 (3)	C4—C5—N1—C6	−0.1 (3)
N2—C6—C7—C8	−178.94 (15)	N4—N3—N2—C9	−0.2 (2)
C6—C7—C8—C4	−0.1 (3)	N4—N3—N2—C6	178.13 (15)
C5—C4—C8—C7	0.0 (3)	C10—C9—N2—N3	−0.04 (19)
C3—C4—C8—C7	−178.31 (16)	C10—C9—N2—C6	−178.15 (16)
N2—C9—C10—N4	0.24 (19)	N1—C6—N2—N3	−171.57 (14)
N2—C9—C10—C11	178.12 (17)	C7—C6—N2—N3	7.5 (2)
C9—C10—C11—C13	155.10 (19)	N1—C6—N2—C9	6.4 (3)
N4—C10—C11—C13	−27.3 (3)	C7—C6—N2—C9	−174.54 (16)
C9—C10—C11—C12	−135.4 (2)	O2—C3—O1—C2	−2.2 (3)
N4—C10—C11—C12	42.2 (3)	C4—C3—O1—C2	176.58 (15)
C10—C11—C12—C13	−110.0 (2)	C1—C2—O1—C3	−178.22 (16)

### Hydrogen-bond geometry (Å, º)

**Table:**

*D*—H···*A*	*D*—H	H···*A*	*D*···*A*	*D*—H···*A*
C5—H5···N1^i^	0.93	2.60	3.375 (2)	141
C7—H7···N3^ii^	0.93	2.42	3.245 (2)	147
C9—H9···O2^i^	0.93	2.54	3.422 (2)	159

Symmetry codes: (i) −*x*+1, −*y*, −*z*+1; (ii) −*x*+2, −*y*+1, −*z*+1.
